# Characteristics of Posterior Corneal Astigmatism in 28,551 Chinese Eyes with Cataracts

**DOI:** 10.3390/jcm15218442

**Published:** 2026-10-29

**Authors:** Yushen Xu, Wenwen He, Xiangjia Zhu

**Affiliations:** 1 Department of Ophthalmology, Shanghai Eye and ENT Hospital, Fudan University, Shanghai 200031, China; 2 NHC Key Laboratory of Myopia, Fudan University, Shanghai 200031, China; 3 Key Laboratory of Myopia, Chinese Academy of Medical Science, Shanghai 200031, China; 4 Shanghai Key Laboratory of Visual Impairment and Restoration, Shanghai 200031, China

**Keywords:** posterior corneal astigmatism, cataract, corneal biometry, preoperative assessment

## Abstract

**Background/Objectives**: Posterior corneal astigmatism (PCA) varies among individuals, potentially limiting the applicability of population-based estimates. This study aimed to characterize PCA and evaluate high-magnitude PCA and horizontal posterior steep-meridian orientation. **Methods**: Cataract patients undergoing preoperative biometry with the IOLMaster 700 were assessed for PCA magnitude and orientation. Factors associated with high-magnitude PCA (≥0.50 D) and horizontal orientation were evaluated. Fellow-eye concordance and vector discrepancies between Abulafia–Koch (AK) estimates and measured corneal astigmatism were analyzed. **Results**: The study included 28,551 patients (28,551 eyes). PCA magnitude was between 0.10 and <0.40 D in 76.6% of eyes, and 91.7% had a vertical posterior steep meridian. High-magnitude PCA and horizontal orientation occurred in 5.1% and 2.5% of eyes. Across PCA magnitude groups, the largest differences involved anterior corneal astigmatism (ACA) magnitude (ε^2^ = 0.090), total corneal astigmatism (TCA) magnitude (ε^2^ = 0.047), and age (ε^2^ = 0.036). Across orientation groups, ACA and TCA magnitude showed the largest differences (ε^2^ = 0.044 and 0.049). High-magnitude PCA was independently associated with younger age, the female sex, a larger ACA magnitude, and with-the-rule (WTR) ACA. Horizontal orientation was associated with older age, a larger ACA magnitude, against-the-rule (ATR) or oblique ACA, and smaller white-to-white distance (WTW). Discrepancies between AK estimates and measured PCA and TCA were greater in both subgroups, with TCA discrepancies exceeding 0.50 D in 40.6% of high-magnitude and 40.4% of horizontal-orientation eyes. **Conclusions**: Most eyes followed the conventional PCA pattern, whereas high-magnitude PCA and horizontal posterior steep-meridian orientation showed distinct profiles and greater discrepancies from AK estimates. Population-based estimates may therefore be less representative in these eyes.

## 1. Introduction

Cataract surgery has evolved from a purely sight-restoring procedure into a refractive intervention in which postoperative visual quality, refractive precision, and spectacle independence are increasingly emphasized [1]. Pre-existing corneal astigmatism is common in cataract patients and remains a major source of residual postoperative refractive error if not adequately characterized and managed [1,2]. Accurate assessment of corneal astigmatism is therefore essential for toric intraocular lens (IOL) selection and optimization of refractive outcomes [3]. Even relatively low levels of residual astigmatism can compromise uncorrected visual outcomes after cataract surgery [4].

Surgical planning has traditionally relied on anterior corneal astigmatism (ACA), but posterior corneal astigmatism (PCA) also contributes to total corneal astigmatism (TCA) [5,6]. Koch et al. reported that PCA magnitude is typically about 0.30 D and that the posterior steep meridian is vertically oriented in most eyes. Failure to account for PCA may therefore lead to systematic misestimation of TCA [6]. Unlike the age-related shift in ACA from with-the-rule (WTR) to against-the-rule (ATR) orientation, PCA generally retains an ATR refractive effect and shows relatively limited age-related changes in orientation [5,7,8,9]. However, large-scale studies in Chinese eyes with cataracts remain limited, particularly those characterizing less common but potentially relevant PCA patterns.

Previous studies have described the overall distribution of PCA and examined its effect on TCA estimation and toric IOL calculations [8,10]. A high PCA magnitude and horizontal posterior steep-meridian orientation represent departures from the conventional posterior corneal profile in magnitude and direction, respectively. How well population-based PCA estimates represent these less common patterns remains unclear [11,12]. Their prevalence, associated biometric characteristics, fellow-eye concordance, and discrepancies between regression-based estimates and measured corneal astigmatism remain incompletely understood.

Therefore, using a hospital-based dataset of 28,551 Chinese eyes with cataracts, we aimed to characterize the magnitude and posterior steep-meridian orientation distributions of PCA and to examine two less common patterns, namely high-magnitude PCA and horizontal posterior steep-meridian orientation. We further evaluated fellow-eye concordance and compared Abulafia–Koch estimates with measured PCA and TCA across these subgroups.

## 2. Materials and Methods

### 2.1. Ethics

This study was approved by the institutional ethics committee (Approval No. 2020068) and adhered to the tenets of the Declaration of Helsinki. Written informed consent for the use of clinical data was obtained from all participants before cataract surgery.

### 2.2. Study Design and Participants

This retrospective, hospital-based, cross-sectional study included adults with cataracts who underwent routine preoperative assessment at a tertiary ophthalmic hospital in Shanghai, China, between August 2022 and December 2024. All patients underwent Scheimpflug corneal tomography (Pentacam, Oculus Optikgeräte GmbH) to screen for corneal diseases, including keratoconus and other corneal ectatic disorders. Pentacam-derived measurements were not included in the analyses. Patients younger than 20 years and eyes lacking complete, good-quality biometry were excluded. Additional exclusion criteria included keratoconus or other corneal ectatic disorders, previous ocular surgery, corneal pathology affecting keratometry, poor fixation, unmeasurable keratometry, and device-reported quality warnings. At least two independent preoperative examinations were available for each included eye. Consistency of ACA, PCA, and TCA across examinations was assessed using the maximum pairwise astigmatic vector difference, calculated as $2\sqrt{{\left(∆J0\right)}^{2}+{\left(∆J45\right)}^{2}}$. Eyes showing marked between-examination discrepancies were excluded. The latest eligible examination was retained for each eye, and one eye per patient was randomly selected for primary analysis. For fellow-eye analyses, the selected eye served as the index eye, and the latest eligible examination of the fellow eye was used.

### 2.3. Biometric Assessment

All ocular biometric parameters included in the analyses were obtained using the swept-source optical coherence tomography-based IOLMaster 700 (software version 1.80; Carl Zeiss Meditec, Jena, Germany). These included ACA, PCA, and TCA magnitudes and their steep-meridian orientations. Other measurements included axial length (AL), mean keratometry (Km), anterior chamber depth (ACD), lens thickness (LT), central corneal thickness (CCT), and white-to-white distance (WTW).

Certified technicians performed all examinations under standard room illumination according to the manufacturer’s protocol. Fixation and alignment were checked on the full-eye scan image, and measurements with poor fixation, implausible values, or device-reported quality warnings were excluded.

### 2.4. Variable Definitions

PCA magnitude was defined as the absolute value of the signed device-reported PCA. Eyes were categorized as having low-magnitude (<0.30 D), moderate-magnitude (0.30 to <0.50 D), or high-magnitude (≥0.50 D) PCA. The 0.30 D boundary reflects the typical PCA magnitude reported in cataract populations [6,7,8], while the 0.50 D boundary identifies larger posterior astigmatism and is consistent with thresholds used in previous studies of PCA [9,13,14]. The PCA magnitude categories were defined before analysis based on these published findings.

Corneal astigmatism orientation was described by the steep meridian rather than by a cylinder axis [15]. The posterior steep meridian was categorized as vertical (60–120°, inclusive), horizontal (0–30° or 150–180°, inclusive), or oblique (>30° to <60° or >120° to <150°) [6,7]. ACA orientation was classified by the anterior steep meridian as WTR, ATR, or oblique using the same vertical, horizontal, and oblique angular intervals, respectively [7]. Because astigmatic meridians have 180° periodicity, orientation distributions were visualized using double-angle transformation based on absolute PCA magnitude and the posterior steep meridian [16,17].

### 2.5. Statistical Analysis

Continuous and categorical variables are presented as the mean ± standard deviation (SD) and number (percentage), respectively. Proportions of PCA magnitude and posterior steep-meridian orientation categories were reported with 95% confidence intervals (CIs), calculated using the Wilson score method. Associations of PCA magnitude with age and AL were assessed using Spearman correlation.

Group comparisons used Kruskal–Wallis or chi-square tests, as appropriate. Effect sizes were quantified using epsilon-squared and Cramér’s V. *p* values were adjusted using the Benjamini–Hochberg procedure. Significant omnibus tests were followed by post hoc comparisons. Post hoc comparisons used Dunn or chi-square tests with Holm adjustment.

For double-angle plots, PCA magnitude *M* and posterior steep-meridian orientation *θ* were converted to Cartesian components as *x* = *M* cos(2*θ*) and *y* = *M* sin(2*θ*). Centroids were obtained by averaging the two components, with magnitude calculated as $\sqrt{{\bar{x}}^{2}+{\bar{y}}^{2}}$ and orientation as $\frac{1}{2}\mathrm{atan}2(\bar{y},\bar{x})$, normalized to 0–180°. Centroids and vector SDs were calculated overall and by age, AL, and orientation [16,17]. Univariable logistic regression was used to screen candidate covariates (*p* < 0.05), followed by selection based on clinical relevance and redundancy among correlated measures. The high-magnitude PCA model included age, sex, ACA magnitude, ACA orientation, ACD, and WTW; the horizontal-orientation model included the same covariates except sex. Continuous covariates were entered as linear terms on their original scales; male sex and WTR ACA served as categorical reference groups. Analyses used complete cases without imputation. Collinearity was assessed using degree-of-freedom-adjusted generalized variance inflation factors (GVIFs). Adjusted odds ratios (ORs) and 95% CIs were reported. We further examined the distribution of PCA magnitude within the horizontal-orientation group. Sensitivity analyses repeated the horizontal-orientation model in eyes with PCA magnitudes ≥0.10 D and ≥0.20 D, retaining the same covariates. Each threshold was applied to all eyes regardless of posterior steep-meridian orientation.

Fellow-eye analyses evaluated posterior steep-meridian concordance. Horizontal-meridian concordance was assessed using Fisher’s exact test, with sensitivity analyses restricted to horizontal index eyes with PCA magnitudes of at least 0.10, 0.20, and 0.30 D.

For comparisons of measured and Abulafia–Koch (AK)-estimated astigmatism, power-vector components were calculated as $J0=-\frac{C}{2}\sin(2\alpha )$ and $J45=-\frac{C}{2}\sin(2\alpha )$, where *C* is the signed cylinder power and *α* is the corresponding cylinder axis [16,17,18]. The AK-predicted total corneal astigmatism was calculated as J0_AK−TCA_ = 0.75 × J0_ACA_ − 0.085 and J45_AK−TCA_ = 0.75 × J45_ACA_; the AK-implied PCA vector was then obtained by subtracting the measured ACA. The absolute vector discrepancies between AK-implied and measured PCA and between AK-predicted and measured TCA were calculated from the differences in their J0 and J45 components: $2\sqrt{{∆J0}^{2}+{∆J45}^{2}}$. Discrepancies were compared across PCA subgroups using Kruskal–Wallis and Holm-adjusted Dunn tests.

All tests were two-sided, with *p* < 0.05 considered statistically significant after applicable multiplicity adjustment. Analyses were performed using R, version 4.4.2 (R Foundation for Statistical Computing, Vienna, Austria).

## 3. Results

### 3.1. Baseline Characteristics and Overall Distribution of Posterior Corneal Astigmatism

Baseline demographic and ocular biometric characteristics of the study population are summarized in Table 1. A total of 28,551 eyes from 28,551 patients were included, with a mean age of 63.1 ± 12.8 years. The study included 16,100 female patients (56.4%). Mean ACA and TCA magnitudes were 0.93 ± 0.67 D and 0.98 ± 0.68 D, respectively. The mean signed device-reported PCA was −0.27 ± 0.13 D.

**Figure 1:**
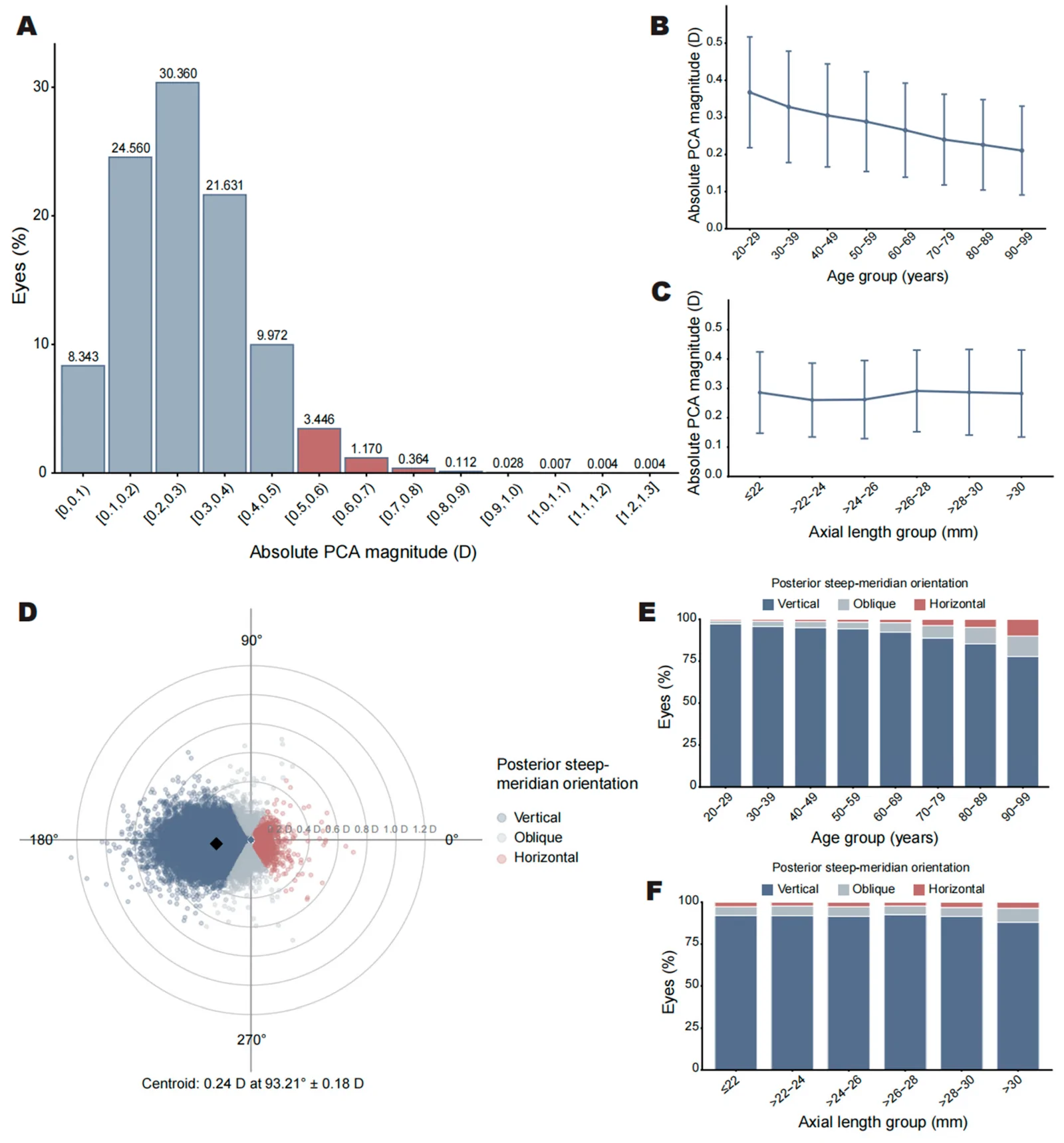
Distribution of posterior corneal astigmatism magnitude and posterior steep-meridian orientation. (**A**) Distribution of absolute posterior corneal astigmatism (PCA) magnitude; red bars indicate a PCA magnitude ≥0.50 diopters (**D**). (**B**) Mean PCA magnitude by age group. (**C**) Mean PCA magnitude by axial length (AL) group. Error bars in panels (**B**,**C**) represent standard deviations. (**D**) Double-angle plot of PCA magnitude and posterior steep-meridian orientation; the black diamond indicates the centroid. (**E**) Distribution of posterior steep-meridian orientation by age group. (**F**) Distribution of posterior steep-meridian orientation by AL group. Colors indicate vertical, oblique, and horizontal posterior steep-meridian orientations.

The overall distribution of PCA magnitude and posterior steep-meridian orientation is shown in Figure 1. PCA magnitude was between 0.10 and <0.40 D in 21,870 eyes (76.6%), with 8668 eyes (30.4%) in the 0.20 to <0.30 D interval (Figure 1A). Low-, moderate-, and high-magnitude PCA occurred in 18,058 eyes (63.25%; 95% CI, 62.69–63.81%), 9027 eyes (31.62%; 95% CI, 31.08–32.16%), and 1466 eyes (5.13%; 95% CI, 4.88–5.40%), respectively. Posterior steep-meridian orientation was vertical in 26,188 eyes (91.72%; 95% CI, 91.40–92.04%), oblique in 1648 eyes (5.77%; 95% CI, 5.51–6.05%), and horizontal in 715 eyes (2.50%; 95% CI, 2.33–2.69%). The overall double-angle centroid was 0.24 D at 93.21°, with a vector SD of 0.18 D (Figure 1D). Centroids were 0.26 D at 92.7°, 0.07 D at 125.6°, and 0.12 D at 175.4° for the vertical, oblique, and horizontal groups, respectively, with vector SDs of 0.16, 0.16, and 0.12 D (Figure S1C).

The mean PCA magnitude decreased across age groups, from 0.37 ± 0.15 D in the 20–29-year group to 0.21 ± 0.12 D in the 90–99-year group (Figure 1B). PCA magnitude was inversely correlated with age (Spearman ρ = −0.22, *p* < 0.001). Vertical posterior steep-meridian orientation was less frequent in older age groups, whereas oblique and horizontal orientations were more frequent (Figure 1E). Age-stratified double-angle plots showed lower centroid magnitudes in older age groups, ranging from 0.34 to 0.16 D, while centroid orientations ranged between 92.6° and 93.9° (Figure S1A).

Across AL groups, the mean absolute PCA magnitude ranged from 0.26 to 0.29 D (Figure 1C). The PCA magnitude showed a negligible correlation with AL (Spearman ρ = 0.03, *p* < 0.001). Posterior steep-meridian distributions were similar across AL groups, although nonvertical orientations were slightly more frequent with ALs > 30 mm (Figure 1F). AL-stratified double-angle plots showed centroid magnitudes of 0.23 to 0.26 D and centroid orientations of 92.7° to 93.8° (Figure S1B).

### 3.2. Characteristics Across PCA Magnitude and Posterior Steep-Meridian Orientation Groups

Significant differences were observed across PCA magnitude groups for all demographic and ocular biometric variables shown in Table 2 after false discovery rate adjustment (all *p* < 0.001). The largest differences were observed for ACA magnitude (ε^2^ = 0.090), TCA magnitude (ε^2^ = 0.047), and age (ε^2^ = 0.036). Mean age decreased across the low-, moderate-, and high-magnitude groups (64.9 ± 12.0, 60.7 ± 13.2, and 56.2 ± 15.0 years, respectively). The proportion of female patients increased from 55.2% to 58.1% and 61.1%, respectively. The mean ACA magnitude increased from 0.814 ± 0.55 D to 0.968 ± 0.60 D and 2.105 ± 1.05 D. Differences in AL, Km, ACD, CCT, LT, and WTW were small (ε^2^ ≤ 0.010). Age, sex distribution, and ACA magnitude differed between all PCA magnitude groups. TCA magnitude was higher in the high-magnitude group, with no difference between the low- and moderate-magnitude groups (Supplementary Table S1).

Across posterior steep-meridian orientation groups, significant differences were observed for age, ACA, TCA, AL, ACD, LT, and WTW, whereas sex, Km, and CCT were comparable among groups (Table 3). The largest differences were observed for TCA magnitude (ε^2^ = 0.049) and ACA magnitude (ε^2^ = 0.044). Mean age increased across the vertical, oblique, and horizontal groups (62.7 ± 12.8, 67.2 ± 11.4, and 68.4 ± 11.4 years, respectively). The mean ACA magnitude also increased from 0.885 ± 0.63 D to 1.246 ± 0.71 D and 1.781 ± 0.92 D, respectively. The mean TCA magnitude similarly increased from 0.926 ± 0.64 D to 1.377 ± 0.78 D and 1.921 ± 0.98 D. Eyes with a horizontal posterior steep meridian also had a smaller mean WTW than those with vertical or oblique orientations. Other biometric differences were small (ε^2^ ≤ 0.003). Pairwise comparisons showed significant differences among the vertical, oblique, and horizontal groups in age, ACA magnitude, and TCA magnitude (Supplementary Table S1).

High-magnitude PCA and a horizontal posterior steep meridian showed limited overlap. A vertical posterior steep meridian was present in 1441 eyes with high-magnitude PCA (98.3%), whereas 671 horizontal-orientation eyes (93.8%) had low-magnitude PCA. Only 11 eyes had both features, accounting for 1.5% of the horizontal group and <0.1% of the overall population (Supplementary Table S2). Within the horizontal-orientation group, the median PCA magnitude was 0.12 D (IQR, 0.08–0.18 D), and 589 eyes (82.4%) had a PCA magnitude < 0.20 D (Supplementary Table S5A). Given the predominance of low-magnitude PCA in the horizontal group, fellow-eye concordance was further examined. Fellow-eye horizontal orientation occurred in 135 of 439 horizontal index eyes (30.8%) and 338 of 17,468 non-horizontal index eyes (1.9%) (OR, 22.49; 95% CI, 17.74–28.48; *p* < 0.001; Supplementary Table S3). Concordance increased to 13 of 28 eyes (46.4%) when index-eye PCA magnitude was ≥0.30 D (Supplementary Table S4).

### 3.3. Factors Associated with High PCA Magnitude and Horizontal Posterior Steep-Meridian Orientation

**Figure 2:**
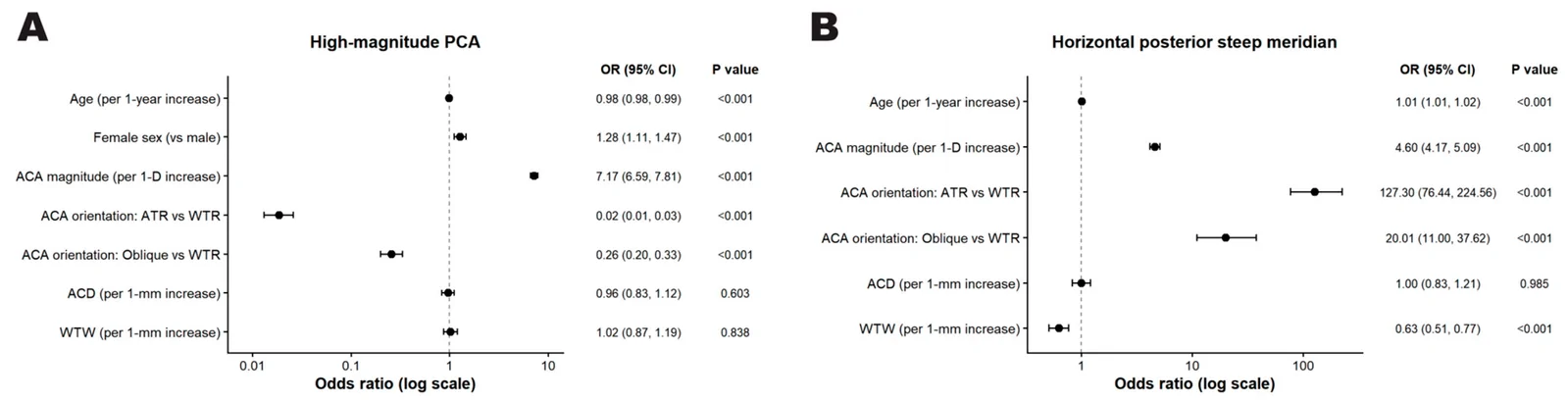
Multivariable logistic regression analyses of factors associated with posterior corneal astigmatism subgroups. (**A**) Factors associated with high-magnitude posterior corneal astigmatism (PCA; ≥0.50 diopters). (**B**) Factors associated with horizontal posterior steep-meridian orientation. Model A included age, sex, ACA magnitude, ACA orientation, ACD, and WTW; Model B included the same covariates except sex. ORs for continuous covariates are expressed per 1-year increase in age, 1-D increase in ACA magnitude, and 1 mm increase in ACD or WTW. The reference categories were male sex and WTR ACA, as applicable. Points and horizontal bars represent adjusted odds ratios (ORs) and 95% confidence intervals (CIs), respectively; the vertical dashed line indicates an OR of 1. ACA = anterior corneal astigmatism; ACD = anterior chamber depth; ATR = against-the-rule; WTR = with-the-rule; WTW = white-to-white distance.

To determine which factors remained independently associated with each PCA phenotype after adjustment for other covariates, multivariable logistic regression analyses were performed. High-magnitude PCA was independently associated with younger age (OR, 0.98; 95% CI, 0.98–0.99), the female sex (OR, 1.28; 95% CI, 1.11–1.47), and a larger ACA magnitude (OR, 7.17; 95% CI, 6.59–7.81; all *p* < 0.001) (Figure 2A). Compared with WTR ACA, ATR (OR, 0.02; 95% CI, 0.01–0.03) and oblique ACA (OR, 0.26; 95% CI, 0.20–0.33) were associated with lower odds of high-magnitude PCA (both *p* < 0.001). ACD and WTW were not independently associated with high-magnitude PCA.

Horizontal orientation was independently associated with older age (OR, 1.01; 95% CI, 1.01–1.02), a larger ACA magnitude (OR, 4.60; 95% CI, 4.17–5.09), and a smaller WTW (OR, 0.63; 95% CI, 0.51–0.77; all *p* < 0.001) (Figure 2B). Compared with WTR ACA, ATR (OR, 127.30; 95% CI, 76.44–224.56) and oblique ACA (OR, 20.01; 95% CI, 11.00–37.62) were associated with higher odds of horizontal orientation (both *p* < 0.001). ACD was not independently associated with this orientation. Degree-of-freedom-adjusted GVIFs were ≤1.15 in both models, indicating no substantial collinearity among the retained covariates. Restricting the cohort to PCA magnitudes ≥0.10 D and ≥0.20 D retained 26,169 and 19,157 eyes, including 444 and 126 horizontal-orientation eyes, respectively. Associations with larger a ACA magnitude, ATR or oblique ACA, and a smaller WTW persisted at both thresholds (all *p* < 0.001). The age association remained significant at ≥0.10 D but not at ≥0.20 D (*p* = 0.981), while ACD remained nonsignificant in both analyses (Supplementary Table S5B).

### 3.4. Discrepancy Between Abulafia–Koch Estimates and Measured Corneal Astigmatism

We next examined whether the discrepancies between AK estimates and measured corneal astigmatism varied according to PCA magnitude and posterior steep-meridian orientation (Table 4). The overall absolute vector discrepancy between AK-implied and measured PCA was 0.181 ± 0.117 D, with a median of 0.158 D (IQR, 0.097–0.240 D). PCA discrepancies exceeded 0.25 D in 6466 eyes (22.6%) and 0.50 D in 490 eyes (1.7%). The corresponding discrepancy between AK-predicted and measured TCA was 0.289 ± 0.194 D, with a median of 0.245 D (IQR, 0.150–0.381 D). TCA discrepancies exceeded 0.25 D in 13,946 eyes (48.8%) and 0.50 D in 3570 eyes (12.5%).

PCA discrepancy differed across the low-, moderate-, and high-magnitude groups (0.177 ± 0.114, 0.180 ± 0.114, and 0.238 ± 0.154 D, respectively; *p* < 0.001; ε^2^ = 0.008). The high-magnitude group differed from both other groups (both *p* < 0.001). Although the low- and moderate-magnitude groups also differed statistically (*p* = 0.007), their mean values differed by only 0.003 D. PCA discrepancy also increased from 0.177 ± 0.113 D in the vertical group to 0.216 ± 0.136 D in the oblique group and 0.257 ± 0.162 D in the horizontal group (*p* < 0.001, ε^2^ = 0.012). All three orientation groups differed significantly from one another (all *p* < 0.001).

Similar patterns were observed for TCA. Mean TCA discrepancies were 0.278 ± 0.182 D, 0.280 ± 0.180 D, and 0.476 ± 0.293 D in the low-, moderate-, and high-magnitude PCA groups, respectively (*p* < 0.001, ε^2^ = 0.027). The high-magnitude group differed from both other groups (both *p* < 0.001), whereas the low- and moderate-magnitude groups did not differ significantly (*p* = 0.053). TCA discrepancy increased across the vertical, oblique, and horizontal groups (0.279 ± 0.186, 0.361 ± 0.224, and 0.473 ± 0.277 D, respectively; *p* < 0.001; ε^2^ = 0.022). All pairwise comparisons were significant (all *p* < 0.001). TCA discrepancies exceeded 0.50 D in 595 of 1466 high-magnitude eyes (40.6%) and 289 of 715 horizontal-orientation eyes (40.4%).

## 4. Discussion

Posterior corneal astigmatism contributes to TCA and is relevant to toric intraocular lens planning [1,6,11,19]. In 28,551 Chinese eyes with cataracts, we characterized PCA distribution and evaluated high-magnitude PCA and horizontal posterior steep-meridian orientation. Most eyes had modest PCA and a vertical posterior steep meridian. However, high-magnitude PCA and horizontal posterior steep-meridian orientation were identified in 5.13% and 2.50% of eyes, respectively. Thus, PCA varies in both magnitude and orientation despite its generally conventional pattern.

Our findings agree with previous Chinese studies reporting PCA magnitudes near 0.3 D and predominantly vertical posterior steep meridians [8,9]. Similar patterns have been reported in Japanese, Korean, and Thai populations [5,7,20,21]. Egyptian and European studies have reported larger PCA magnitudes or fewer vertical posterior steep meridians [14,22,23]. Such differences may reflect population characteristics, age, refractive status, inclusion criteria, or measurement platforms. Direct comparisons have also identified differences in posterior keratometry and astigmatic vector components between IOLMaster 700 and Pentacam, indicating that measurements from these devices should not be assumed to be interchangeable [24,25]. In the present study, older age was associated with lower PCA magnitude and a higher frequency of nonvertical posterior steep meridians. Together with the greater prevalence of ATR ACA in older age groups, these findings suggest age-associated differences in anterior–posterior astigmatic alignment [5,7,20,21,26]. The stronger inverse association with age than previously reported may reflect differences in age distribution and patient selection [5,26]. AL showed only a negligible correlation with PCA magnitude, and PCA magnitude and posterior steep-meridian distributions were generally similar across AL groups [8,9,27,28]. Although the broad AL range reflects the biometric heterogeneity of this cataract cohort, these findings suggest that this heterogeneity was not accompanied by marked differences in the overall PCA distribution.

High-magnitude PCA was associated with younger age, the female sex, larger ACA, and WTR ACA. Importantly, 98.3% of these eyes retained a vertical posterior steep meridian. Thus, high-magnitude PCA primarily represented amplification of the usual vertical pattern. Its association with larger WTR ACA supports previous evidence of stronger anterior–posterior coupling in WTR eyes [7,8,9]. Clinically, large WTR ACA may be accompanied by a large vertically oriented posterior component. This posterior component exerts an ATR refractive effect and partially offsets anterior WTR astigmatism. Assessment based on ACA alone may overestimate the WTR component of TCA [6,19,29].

Horizontal orientation represented a departure from the conventional posterior corneal pattern. It was associated with older age, a larger ACA magnitude, ATR or oblique ACA, and a smaller WTW. The associations with age and ACA orientation may reflect differences in anterior–posterior alignment across age groups [5,7,20,21,26]. Most horizontal-orientation eyes had low-magnitude PCA, and only 11 also had high-magnitude PCA, indicating distinct patterns of variation. At low magnitudes, small vector changes can alter categorical orientation [16,17]. However, horizontal orientation was more frequent in fellow eyes of horizontal index eyes. Concordance persisted when the index-eye PCA was ≥0.30 D. Associations with ACA magnitude, ACA orientation, and WTW persisted after excluding low PCA values, suggesting that these relationships were not confined to eyes with near-zero PCA. In contrast, the age association was no longer significant at the ≥0.20 D threshold. This attenuation may reflect the weak age association and reduced precision in the smaller restricted cohort, although changes in cohort composition may also contribute. Nevertheless, measurement variability may still influence posterior steep-meridian classification, particularly at low PCA magnitudes, even after screening for consistency across repeated examinations. Population-based PCA models may inadequately represent uncommon posterior orientations [11,19,30].

High-magnitude PCA and horizontal posterior steep-meridian orientation deviate from the conventional posterior corneal profile in magnitude and direction, respectively. Both patterns may be less well represented by population-based estimates. Using the AK regression as an example, we found greater discrepancies from measured PCA and TCA in both groups. The AK regression estimates TCA from the average relationship between anterior and total corneal astigmatism, and has improved toric IOL calculations in general cataract populations [18]. Our findings extend previous large-scale evidence of individual differences between AK-based and measured corneal astigmatism by showing greater discrepancies in eyes with high-magnitude PCA or a horizontal posterior steep-meridian orientation [31]. Previous clinical studies suggest that PCA magnitude and orientation may affect toric IOL prediction accuracy [11,12]. However, measured PCA has not consistently outperformed predicted PCA across formulas and devices [19,30]. Our results therefore identify subgroups in which population-based estimates may diverge more from measured corneal astigmatism, rather than demonstrating the superiority of either approach. The clinical relevance of these preoperative vector discrepancies remains to be established, as postoperative refractive outcomes were not assessed in this study.

In summary, our findings delineate both the regularity and the boundaries of population-level PCA patterns in Chinese eyes with cataracts. Although these patterns provide a useful basis for estimation, they do not represent individual corneal geometry equally well in all eyes. Our findings do not support a universal preference for measured or predicted PCA, but provide a framework for recognizing when population-based assumptions merit closer scrutiny. Further studies incorporating postoperative refractive outcomes are needed to determine how this information can best inform toric IOL planning.

## Figures and Tables

**Table 1:** Baseline demographic and ocular biometric characteristics of the study population.

Variable	Overall
Age (years)	63.1 ± 12.8 (20–99)
Sex, *n* (%)	
Male	12,451 (43.6%)
Female	16,100 (56.4%)
Eye, *n* (%)	
OD	14,041 (49.2%)
OS	14,510 (50.8%)
ACA (D)	0.93 ± 0.67 (0.00–6.00)
TCA (D)	0.98 ± 0.68 (0.00–5.95)
PCA (D)	−0.27 ± 0.13 (−1.25–0.00)
AL (mm)	24.54 ± 2.38 (14.90–36.37)
Km (D)	43.94 ± 1.57 (34.86–52.30)
ACD (mm)	3.06 ± 0.47 (1.01–4.50)
CCT (μm)	541 ± 34 (405–650)
LT (mm)	4.51 ± 0.45 (2.54–6.00)
WTW (mm)	11.73 ± 0.44 (10.50–13.48)

Data are presented as the mean ± standard deviation (range) for continuous variables and number (percentage) for categorical variables. ACA and TCA are reported as device-measured astigmatic magnitudes. PCA is reported as the signed device-measured value in the baseline table; magnitude-based analyses used its absolute value. OD, right eye; OS, left eye; ACA, anterior corneal astigmatism; TCA, total corneal astigmatism; PCA, posterior corneal astigmatism; AL, axial length; Km, mean keratometry; ACD, anterior chamber depth; CCT, central corneal thickness; LT, lens thickness; WTW, white-to-white distance.

**Table 2:** Comparison of demographic and ocular biometric characteristics across PCA magnitude subgroups.

Variable	Low-Magnitude PCA (*N* = 18,058)	Moderate-Magnitude PCA (*N* = 9027)	High-Magnitude PCA (*N* = 1466)	Effect Size	FDR-Adjusted *p* Value
Age (years)	64.9 ± 12.0 (20–99)	60.7 ± 13.2 (20–93)	56.2 ± 15.0 (20–93)	ε^2^ = 0.036	<0.001
Sex, *n* (%)				V = 0.035	<0.001
Male	8096 (44.8%)	3784 (41.9%)	571 (38.9%)		
Female	9962 (55.2%)	5243 (58.1%)	895 (61.1%)		
ACA (D)	0.814 ± 0.55 (0.00–5.16)	0.968 ± 0.60 (0.00–5.06)	2.105 ± 1.05 (0.00–6.00)	ε^2^ = 0.090	<0.001
TCA (D)	0.934 ± 0.63 (0.00–5.88)	0.914 ± 0.60 (0.00–5.53)	1.889 ± 1.07 (0.00–5.95)	ε^2^ = 0.047	<0.001
AL (mm)	24.451 ± 2.29 (15.04–36.37)	24.624 ± 2.48 (14.90–35.85)	25.104 ± 2.75 (15.15–34.95)	ε^2^ = 0.003	<0.001
Km (D)	43.849 ± 1.55 (35.53–52.30)	44.039 ± 1.57 (34.86–52.15)	44.385 ± 1.68 (35.97–51.39)	ε^2^ = 0.007	<0.001
ACD (mm)	3.033 ± 0.46 (1.12–4.50)	3.085 ± 0.48 (1.01–4.49)	3.152 ± 0.50 (1.38–4.45)	ε^2^ = 0.005	<0.001
CCT (μm)	541 ± 34 (405–650)	542 ± 33 (405–648)	544 ± 36 (430–645)	ε^2^ = 0.001	<0.001
LT (mm)	4.544 ± 0.44 (2.60–6.00)	4.470 ± 0.46 (2.65–5.97)	4.380 ± 0.48 (2.54–5.86)	ε^2^ = 0.010	<0.001
WTW (mm)	11.705 ± 0.43 (10.50–13.47)	11.766 ± 0.43 (10.52–13.48)	11.793 ± 0.45 (10.55–13.45)	ε^2^ = 0.005	<0.001

Data are presented as the mean ± standard deviation (range) for continuous variables and number (percentage) for categorical variables. PCA magnitude was categorized as low (<0.30 D), moderate (0.30 to <0.50 D), or high (≥0.50 D). Continuous variables were compared using the Kruskal–Wallis test, with epsilon-squared (ε^2^) as the effect size; categorical variables were compared using the chi-square test, with Cramér’s V as the effect size. *p* values were adjusted within the table using the Benjamini–Hochberg false discovery rate procedure. All tests were two-sided. *N*, number of eyes; ACA, anterior corneal astigmatism; TCA, total corneal astigmatism; PCA, posterior corneal astigmatism; AL, axial length; Km, mean keratometry; ACD, anterior chamber depth; CCT, central corneal thickness; LT, lens thickness; WTW, white-to-white distance.

**Table 3:** Comparison of demographic and ocular biometric characteristics across posterior steep-meridian orientation subgroups.

Variable	Vertical Posterior Steep Meridian (*N* = 26,188)	Oblique Posterior Steep Meridian (*N* = 1648)	Horizontal Posterior Steep Meridian (*N* = 715)	Effect Size	FDR-Adjusted *p* Value
Age (years)	62.7 ± 12.8 (20–97)	67.2 ± 11.4 (20–99)	68.4 ± 11.4 (24–94)	ε^2^ = 0.012	<0.001
Sex, *n* (%)				V = 0.006	0.616
Male	11,415 (43.6%)	734 (44.5%)	302 (42.2%)		
Female	14,773 (56.4%)	914 (55.5%)	413 (57.8%)		
ACA (D)	0.885 ± 0.63 (0.00–6.00)	1.246 ± 0.71 (0.00–5.78)	1.781 ± 0.92 (0.00–5.23)	ε^2^ = 0.044	<0.001
TCA (D)	0.926 ± 0.64 (0.00–5.95)	1.377 ± 0.78 (0.00–5.94)	1.921 ± 0.98 (0.00–5.23)	ε^2^ = 0.049	<0.001
AL (mm)	24.526 ± 2.36 (14.90–36.37)	24.625 ± 2.53 (15.11–35.46)	24.805 ± 2.66 (19.09–35.56)	ε^2^ < 0.001	0.015
Km (D)	43.936 ± 1.56 (34.86–52.30)	43.933 ± 1.61 (35.56–50.15)	43.972 ± 1.62 (38.46–50.40)	ε^2^ < 0.001	0.616
ACD (mm)	3.062 ± 0.47 (1.01–4.50)	2.990 ± 0.47 (1.12–4.38)	2.980 ± 0.45 (1.47–4.17)	ε^2^ = 0.002	<0.001
CCT (μm)	542 ± 34 (405–650)	541 ± 35 (408–648)	540 ± 36 (435–641)	ε^2^ < 0.001	0.283
LT (mm)	4.505 ± 0.45 (2.54–6.00)	4.585 ± 0.44 (2.79–5.90)	4.617 ± 0.43 (3.15–5.91)	ε^2^ = 0.003	<0.001
WTW (mm)	11.743 ± 0.43 (10.50–13.48)	11.592 ± 0.42 (10.53–12.95)	11.515 ± 0.43 (10.52–13.03)	ε^2^ = 0.012	<0.001

Data are presented as the mean ± standard deviation (range) for continuous variables and number (percentage) for categorical variables. Posterior steep-meridian orientation was categorized as vertical (60–120°, inclusive), horizontal (0–30° or 150–180°), or oblique (>30° to <60° or >120° to <150°). Continuous variables were compared using the Kruskal–Wallis test, with epsilon-squared (ε^2^) as the effect size; categorical variables were compared using the chi-square test, with Cramér’s V as the effect size. *p* values were adjusted within the table using the Benjamini–Hochberg false discovery rate procedure. All tests were two-sided. *N*, number of eyes; ACA, anterior corneal astigmatism; TCA, total corneal astigmatism; AL, axial length; Km, mean keratometry; ACD, anterior chamber depth; CCT, central corneal thickness; LT, lens thickness; WTW, white-to-white distance.

**Table 4:** Vector discrepancies between Abulafia–Koch estimates and measured corneal astigmatism.

Stratification	Group	Eyes, *n*	Absolute Vector Discrepancy, D Mean ± SD	Median (IQR), D	>0.25 D, *n* (%)	>0.50 D, *n* (%)
**AK-implied PCA vs. measured PCA**						
Overall	All eyes	28,551	0.181 ± 0.117	0.158 (0.097–0.240)	6466 (22.6)	490 (1.7)
PCA magnitude	Low (<0.30 D)	18,058	0.177 ± 0.114	0.155 (0.095–0.235)	3874 (21.5)	274 (1.5)
	Moderate (0.30–<0.50 D)	9027	0.180 ± 0.114	0.159 (0.098–0.238)	2020 (22.4)	132 (1.5)
	High (≥0.50 D)	1466	0.238 ± 0.154	0.206 (0.126–0.322)	572 (39.0)	84 (5.7)
Posterior steep-meridian orientation	Vertical	26,188	0.177 ± 0.113	0.155 (0.095–0.235)	5635 (21.5)	371 (1.4)
	Oblique	1648	0.216 ± 0.136	0.190 (0.119–0.278)	525 (31.9)	67 (4.1)
	Horizontal	715	0.257 ± 0.162	0.223 (0.141–0.343)	306 (42.8)	52 (7.3)
**AK-predicted TCA vs. measured TCA**						
Overall	All eyes	28,551	0.289 ± 0.194	0.245 (0.150–0.381)	13,946 (48.8)	3570 (12.5)
PCA magnitude	Low (<0.30 D)	18,058	0.278 ± 0.182	0.237 (0.146–0.369)	8486 (47.0)	1989 (11.0)
	Moderate (0.30–<0.50 D)	9027	0.280 ± 0.180	0.243 (0.150–0.371)	4368 (48.4)	986 (10.9)
	High (≥0.50 D)	1466	0.476 ± 0.293	0.424 (0.248–0.653)	1092 (74.5)	595 (40.6)
Posterior steep-meridian orientation	Vertical	26,188	0.279 ± 0.186	0.239 (0.147–0.368)	12,375 (47.3)	2904 (11.1)
	Oblique	1648	0.361 ± 0.224	0.318 (0.193–0.476)	1028 (62.4)	377 (22.9)
	Horizontal	715	0.473 ± 0.277	0.429 (0.254–0.644)	543 (75.9)	289 (40.4)

Data are presented as indicated. Absolute vector discrepancy was calculated from the differences in J0 and J45 between the corresponding AK estimate and measured value. AK-implied PCA was derived as AK-predicted TCA minus measured ACA. All overall Kruskal–Wallis comparisons were significant (*p* < 0.001). Epsilon-squared values for PCA magnitude and posterior steep-meridian orientation were 0.008 and 0.012, respectively, for PCA discrepancies and 0.027 and 0.022, respectively, for TCA discrepancies. Pairwise comparisons used Dunn tests with Holm adjustment. For TCA discrepancies, the low- and moderate-magnitude groups did not differ significantly (*p* = 0.053). The centroid discrepancies were 0.057 D at an equivalent steep meridian of 105.0° for PCA and 0.049 D at 108.6° for TCA. AK, Abulafia–Koch; PCA, posterior corneal astigmatism; TCA, total corneal astigmatism; ACA, anterior corneal astigmatism; SD, standard deviation; IQR, interquartile range.

## Data Availability

The data supporting the findings of this study are not publicly available due to patient privacy restrictions, but are available from the corresponding author upon reasonable request and subject to institutional approval.

## Supplementary Materials

The following supporting information can be downloaded at https://www.mdpi.com/article/10.3390/jcm15218442/s1, Figure S1: Double-angle plots of posterior corneal astigmatism stratified by age, axial length, and posterior steep-meridian orientation; Table S1: Pairwise post hoc comparisons of selected characteristics across PCA magnitude and posterior steep-meridian orientation subgroups; Table S2: Cross-classification of posterior corneal astigmatism magnitude and posterior steep-meridian orientation; Table S3: Fellow-eye concordance of posterior steep-meridian orientation; Table S4: Sensitivity analysis of fellow-eye concordance for posterior corneal astigmatism with a horizontal steep meridian; Table S5: PCA magnitude distribution in eyes with a horizontal posterior steep meridian and sensitivity analyses excluding low PCA values. (A) Distribution of PCA magnitude in the horizontal posterior steep-meridian group. (B) Sensitivity analyses of factors associated with horizontal posterior steep-meridian orientation.

## Abbreviations

The following abbreviations are used in this manuscript:

PCA	Posterior corneal astigmatism
ACA	Anterior corneal astigmatism
TCA	Total corneal astigmatism
AK	Abulafia–Koch
WTR	With-the-rule
ATR	Against-the-rule
WTW	White-to-white distance
AL	Axial length
ACD	Anterior chamber depth
LT	Lens thickness
CCT	Central corneal thickness
IOL	Intraocular lens
OR	Odds ratio
CI	Confidence interval
GVIF	Generalized variance inflation factor
