# Supplementary material for: Characteristics of Posterior Corneal Astigmatism in 28,551 Chinese Eyes with Cataracts
Source: J Clin Med. 2026 Oct 29;15(21):8442. doi: 10.3390/jcm15218442 (PMC13763714; doi:10.3390/jcm15218442)
Supplement: Supplementary file 1 [file jcm-15-08442-s001.zip › Figure S1.pdf]

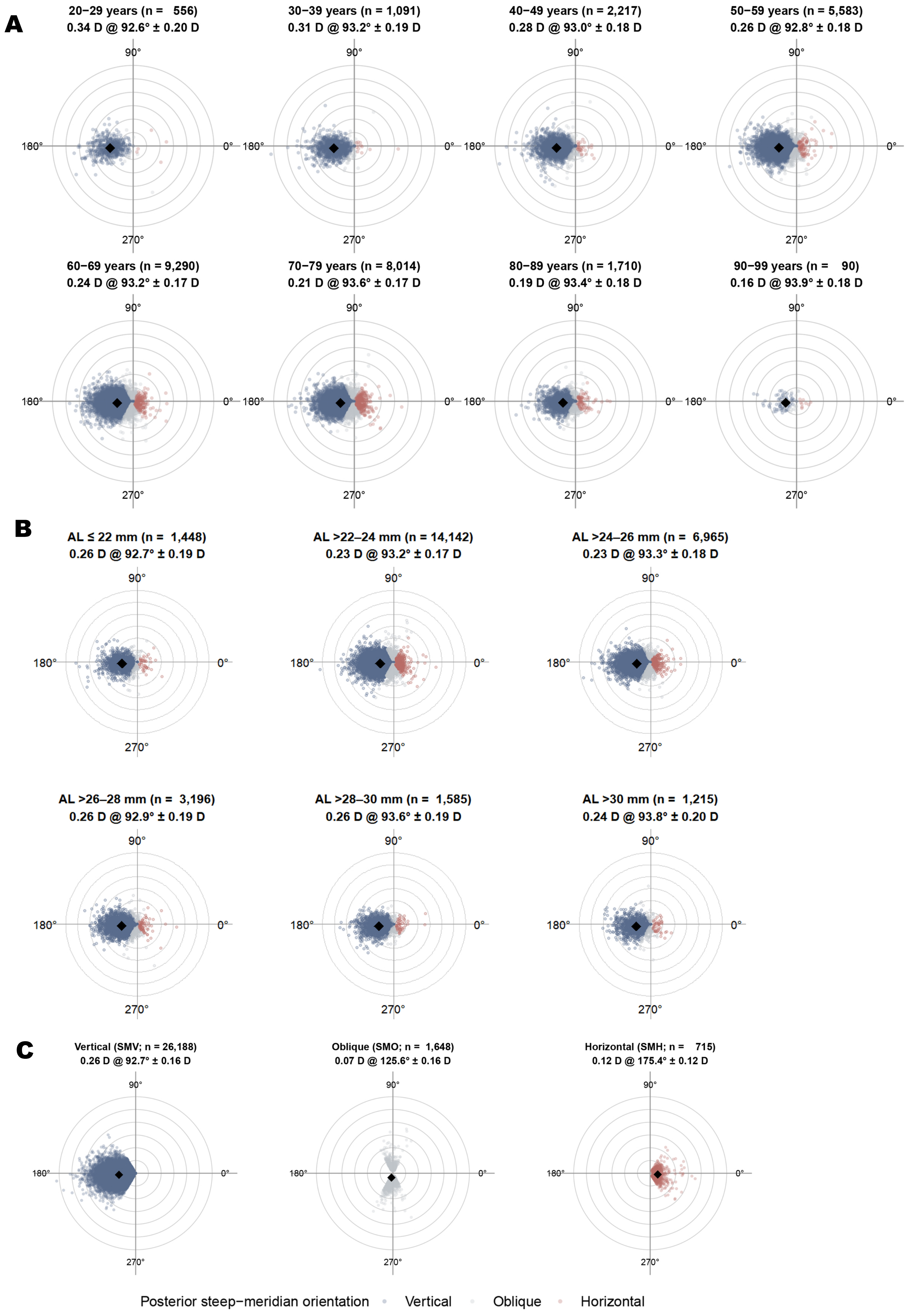
A
20−29 years (n = 556)
0.34 D @ 92.6° ± 0.20 D
30−39 years (n = 1,091)
0.31 D @ 93.2° ± 0.19 D
40−49 years (n = 2,217)
0.28 D @ 93.0° ± 0.18 D
50−59 years (n = 5,583)
0.26 D @ 92.8° ± 0.18 D
60−69 years (n = 9,290)
0.24 D @ 93.2° ± 0.17 D
70−79 years (n = 8,014)
0.21 D @ 93.6° ± 0.17 D
80−89 years (n = 1,710)
0.19 D @ 93.4° ± 0.18 D
90−99 years (n = 90)
0.16 D @ 93.9° ± 0.18 D
B
AL ≤ 22 mm (n = 1,448)
0.26 D @ 92.7° ± 0.19 D
AL >22–24 mm (n = 14,142)
0.23 D @ 93.2° ± 0.17 D
AL >24–26 mm (n = 6,965)
0.23 D @ 93.3° ± 0.18 D
AL >26–28 mm (n = 3,196)
0.26 D @ 92.9° ± 0.19 D
AL >28–30 mm (n = 1,585)
0.26 D @ 93.6° ± 0.19 D
AL >30 mm (n = 1,215)
0.24 D @ 93.8° ± 0.20 D
C
Vertical (SMV; n = 26,188)
0.26 D @ 92.7° ± 0.16 D
Oblique (SMO; n = 1,648)
0.07 D @ 125.6° ± 0.16 D
Horizontal (SMH; n = 715)
0.12 D @ 175.4° ± 0.12 D
90°
180°
0°
270°
Posterior steep−meridian orientation
Vertical
Oblique
Horizontal
