# Supplementary material for: Characteristics of Posterior Corneal Astigmatism in 28,551 Chinese Eyes with Cataracts
Source: J Clin Med. 2026 Oct 29;15(21):8442. doi: 10.3390/jcm15218442 (PMC13763714; doi:10.3390/jcm15218442)
Supplement: Supplementary file 1 [file jcm-15-08442-s001.zip › Supplementary Table S5.pdf]

Supplementary Table S5. PCA magnitude distribution in eyes with a horizontal posterior steep meridian and sensitivity analyses excluding low PCA values

A. Distribution of PCA magnitude in the horizontal posterior steep-meridian group

PCA magnitude (D)	Eyes, n (%)
<0.10	271 (37.90)
0.10–<0.20	318 (44.48)
0.20–<0.30	82 (11.47)
0.30–<0.40	20 (2.80)
0.40–<0.50	13 (1.82)
≥0.50	11 (1.54)

N = 715. Mean ± SD: 0.14 ± 0.10 D; median (IQR): 0.12 (0.08-0.18) D; range: 0.04-0.71 D. Percentages use all 715 horizontal-orientation eyes as the denominator and may not sum to 100% because of rounding.

B. Sensitivity analyses of factors associated with horizontal posterior steep-meridian orientation

PCA magnitude threshold	Total eyes, n	Horizontal, n (%)	Nonhorizontal, n
No restriction	28,551	715 (2.50)	27,836
PCA ≥0.10 D	26,169	444 (1.70)	25,725
PCA ≥0.20 D	19,157	126 (0.66)	19,031

Variable	No restriction<br>Adjusted OR (95% CI)	P value	PCA ≥0.10 D<br>Adjusted OR (95% CI)	P value	PCA ≥0.20 D<br>Adjusted OR (95% CI)	P value
Age (per 1-year increase)	1.012 (1.007–1.017)	<0.001	1.010 (1.005–1.014)	<0.001	1.000 (0.982–1.020)	0.981
ACA magnitude (per 1-D increase)	4.60 (4.17–5.09)	<0.001	5.59 (4.96–6.32)	<0.001	5.44 (4.57–6.54)	<0.001
ACA orientation: ATR vs WTR	127.30 (76.44–224.56)	<0.001	143.73 (79.29–278.78)	<0.001	97.15 (43.71–242.85)	<0.001
ACA orientation: Oblique vs WTR	20.01 (11.00–37.62)	<0.001	18.65 (9.20–39.00)	<0.001	11.32 (4.15–31.50)	<0.001
ACD (per 1-mm increase)	1.00 (0.83–1.21)	0.985	1.03 (0.81–1.32)	0.786	1.06 (0.67–1.67)	0.816
WTW (per 1-mm increase)	0.63 (0.51–0.77)	<0.001	0.51 (0.39–0.67)	<0.001	0.42 (0.26–0.70)	<0.001

The PCA magnitude threshold was applied to all eyes, irrespective of posterior steep-meridian orientation. Each multivariable logistic regression model included age, ACA magnitude, ACA orientation, ACD, and WTW; the outcome was horizontal versus nonhorizontal orientation. WTR was the reference category for ACA orientation. Confidence intervals were calculated using profile likelihood, and P values were obtained from two-sided Wald tests. Age ORs and confidence intervals are shown to three decimal places to preserve precision; other ORs are shown to two decimal places. PCA, posterior corneal astigmatism; ACA, anterior corneal astigmatism; ACD, anterior chamber depth; WTW, white-to-white distance; WTR, with-the-rule; ATR, against-the-rule; OR, odds ratio; CI, confidence interval; SD, standard deviation; IQR, interquartile range.
