# Supplementary material for: Characteristics of Posterior Corneal Astigmatism in 28,551 Chinese Eyes with Cataracts
Source: J Clin Med. 2026 Oct 29;15(21):8442. doi: 10.3390/jcm15218442 (PMC13763714; doi:10.3390/jcm15218442)
Supplement: Supplementary file 1 [file jcm-15-08442-s001.zip › Supplementary Table S1.pdf]

Supplementary Table S1. Pairwise post hoc comparisons of selected characteristics across PCA magnitude and posterior steep-meridian orientation subgroups

Grouping variable	Characteristic	Pairwise comparison	Holm-adjusted P value
PCA magnitude	Age (years)	Low vs Moderate	<0.001
	Age (years)	Low vs High	<0.001
	Age (years)	Moderate vs High	<0.001
	Sex	Low vs Moderate	<0.001
	Sex	Low vs High	<0.001
	Sex	Moderate vs High	0.032
	ACA magnitude (D)	Low vs Moderate	<0.001
	ACA magnitude (D)	Low vs High	<0.001
	ACA magnitude (D)	Moderate vs High	<0.001
	TCA magnitude (D)	Low vs Moderate	0.260
	TCA magnitude (D)	Low vs High	<0.001
	TCA magnitude (D)	Moderate vs High	<0.001
Posterior steep-meridian orientation	Age (years)	Vertical vs Oblique	<0.001
	Age (years)	Vertical vs Horizontal	<0.001
	Age (years)	Oblique vs Horizontal	0.006
	ACA magnitude (D)	Vertical vs Oblique	<0.001
	ACA magnitude (D)	Vertical vs Horizontal	<0.001
	ACA magnitude (D)	Oblique vs Horizontal	<0.001
	TCA magnitude (D)	Vertical vs Oblique	<0.001
	TCA magnitude (D)	Vertical vs Horizontal	<0.001
	TCA magnitude (D)	Oblique vs Horizontal	<0.001

Pairwise comparisons of continuous variables were performed using Dunn’s test following a significant Kruskal-Wallis test. Pairwise comparisons of sex distributions were performed using Pearson chi-square tests. P values were adjusted separately for the three pairwise comparisons of each characteristic using the Holm method. All tests were two-sided. ACA, anterior corneal astigmatism; TCA, total corneal astigmatism; PCA, posterior corneal astigmatism.
