# Supplementary material for: Characteristics of Posterior Corneal Astigmatism in 28,551 Chinese Eyes with Cataracts
Source: J Clin Med. 2026 Oct 29;15(21):8442. doi: 10.3390/jcm15218442 (PMC13763714; doi:10.3390/jcm15218442)
Supplement: Supplementary file 1 [file jcm-15-08442-s001.zip › Supplemental Figure Legends.pdf]

Supplemental Figure Legends

Supplemental Figure S1. Double-angle plots of posterior corneal astigmatism stratified by age, axial length, and posterior steep-meridian orientation. (A) Posterior corneal astigmatism (PCA) distribution by age group. (B) PCA distribution by axial length (AL) group. (C) PCA distribution by posterior steep-meridian orientation. Each point represents an eye, and black diamonds indicate subgroup centroids. Values above each plot show centroid magnitude, centroid orientation, and vector standard deviation. Colors indicate vertical, oblique, and horizontal posterior steep-meridian orientations. D = diopters; SMV = steep-meridian vertical; SMO = steep-meridian oblique; SMH = steep-meridian horizontal.
