# Supplementary material for: Characteristics of Posterior Corneal Astigmatism in 28,551 Chinese Eyes with Cataracts
Source: J Clin Med. 2026 Oct 29;15(21):8442. doi: 10.3390/jcm15218442 (PMC13763714; doi:10.3390/jcm15218442)
Supplement: Supplementary file 1 [file jcm-15-08442-s001.zip › Supplementary Table S2.pdf]

Supplementary Table S2. Cross-classification of posterior corneal astigmatism magnitude and posterior steep-meridian orientation

Posterior steep-meridian orientation	Low-magnitude PCA	Moderate-magnitude PCA	High-magnitude PCA	Total
Vertical	15,834 (60.5)	8,913 (34.0)	1,441 (5.5)	26,188
Oblique	1,553 (94.2)	81 (4.9)	14 (0.8)	1,648
Horizontal	671 (93.8)	33 (4.6)	11 (1.5)	715

Data are presented as n (row percentage). PCA magnitude was categorized as low (<0.30 D), moderate (0.30 to <0.50 D), or high (≥0.50 D). The high-magnitude horizontal subgroup included 11 eyes, representing 1.5% of eyes with a horizontal posterior steep meridian, 0.75% of high-magnitude PCA, and 0.04% of all included eyes. The distribution of PCA magnitude differed across posterior steep-meridian orientation groups ($P < 0.001$, chi-square test). PCA, posterior corneal astigmatism.
