# Supplementary material for: Characteristics of Posterior Corneal Astigmatism in 28,551 Chinese Eyes with Cataracts
Source: J Clin Med. 2026 Oct 29;15(21):8442. doi: 10.3390/jcm15218442 (PMC13763714; doi:10.3390/jcm15218442)
Supplement: Supplementary file 1 [file jcm-15-08442-s001.zip › Supplementary Table S3.pdf]

Supplementary Table S3. Fellow-eye concordance of posterior steep-meridian orientation

Index-eye orientation	Fellow-eye vertical	Fellow-eye oblique	Fellow-eye horizontal	Total
Vertical	15,504 (94.21)	725 (4.41)	227 (1.38)	16,456
Oblique	697 (68.87)	204 (20.16)	111 (10.97)	1,012
Horizontal	203 (46.24)	101 (23.01)	135 (30.75)	439
Total	16,404 (91.61)	1,030 (5.75)	473 (2.64)	17,907

Data are presented as n (row percentage) unless otherwise indicated. Eligible fellow-eye data were available for 17,907 of 28,551 patients. The index eye was the randomly selected eye included in the primary analysis, and the latest eligible measurement was retained for the fellow eye. Vertical was defined as 60°-120° inclusive; horizontal as 0°-30° or 150°-180° inclusive; and oblique as >30° to <60° or >120° to <150°.
