# Supplementary material for: Characteristics of Posterior Corneal Astigmatism in 28,551 Chinese Eyes with Cataracts
Source: J Clin Med. 2026 Oct 29;15(21):8442. doi: 10.3390/jcm15218442 (PMC13763714; doi:10.3390/jcm15218442)
Supplement: Supplementary file 1 [file jcm-15-08442-s001.zip › Supplementary Table S4.pdf]

Supplementary Table S4. Sensitivity analysis of fellow-eye concordance for posterior corneal astigmatism with a horizontal steep meridian

Index-eye PCA magnitude threshold	Index-eye horizontal, n	Fellow-eye horizontal among index-eye horizontal, n (%)	Index-eye non-horizontal, n	Fellow-eye horizontal among index-eye non-horizontal, n (%)	Odds ratio (95% CI)	P value
No restriction	439	135 (30.75)	17,468	338 (1.93)	22.49 (17.74–28.48)	<0.001
≥0.10 D	279	101 (36.20)	17,468	338 (1.93)	28.72 (21.78–37.79)	<0.001
≥0.20 D	87	36 (41.38)	17,468	338 (1.93)	35.73 (22.34–56.59)	<0.001
≥0.30 D	28	13 (46.43)	17,468	338 (1.93)	43.87 (19.05–99.41)	<0.001

Odds ratios, exact 95% confidence intervals, and P values were calculated using Fisher’s exact test. PCA, posterior corneal astigmatism; CI, confidence interval.
